# Combining docking with pharmacophore filtering for improved virtual screening

**DOI:** 10.1186/1758-2946-1-6

**Published:** 2009-05-20

**Authors:** Megan L Peach, Marc C Nicklaus

**Affiliations:** 1 Basic Research Program, SAIC-Frederick, Inc, NCI-Frederick, Frederick, Maryland 21702, USA; 2 Laboratory of Medicinal Chemistry, Center for Cancer Research, National Cancer Institute, Frederick, Maryland 21702, USA

## Abstract

**Background:**

Virtual screening is used to distinguish potential leads from inactive compounds in a database of chemical samples. One method for accomplishing this is by docking compounds into the structure of a receptor binding site in order to rank-order compounds by the quality of the interactions they form with the receptor. It is generally established that docking can be reasonably successful at generating good poses of a ligand in an active site. However, the scoring functions that are used with docking are typically not successful at correctly ranking ligands according to binding affinity or even distinguishing correct poses of a given ligand from incorrect ones.

**Results:**

We have developed a simple method for reducing the number of false positives in a virtual screen, meaning ligands which are scored highly by the docking program but do not bind well in reality. This method uses a docking program for pose generation without regard to scoring, followed by filtering with receptor-based pharmacophore searches. We applied it to three test-case targets: neuraminidase A, cyclin-dependent kinase 2, and the C1 domain of protein kinase C.

**Conclusion:**

The pharmacophore filtering method can perform better than more traditional docking + scoring methods, and allows the advantages of both docking-based and pharmacophore-based approaches to virtual screening to be fully realized.

## Background

The goal of virtual screening is to select, relatively rapidly and cheaply, a small subset of compounds predicted to have activity against a given biological target out of a large database of compounds. While it is possible to screen large databases in their entirety using automated high-throughput screening methods, this is expensive and requires a substantial investment in infrastructure and assay development. The idea of virtual screening is to test compounds computationally in order to reduce the number of compounds to be screened experimentally, with the additional advantage that the number of compounds in the final set can easily be adjusted according to the resources available for assaying.

The database used for virtual screening can be a collection of commercially available compounds, such as ZINC [1] or the ChemNavigator iResearch Library [2], both of which are meta-collections of supplier catalogs. Pharmaceutical companies typically have an in-house database of previously synthesized molecules. A publicly available alternative to this is the open NCI database [3], a collection of compounds that have been tested over the past few decades in the National Cancer Institute's screens for anticancer activity. Small samples from a subset of this collection are available for research purposes upon request [4].

A variety of computational methods can be used for virtual screening depending on the desired size of the final subset and on the amount of information known about the target, its natural ligands, and any known inhibitors. Here we focus on the method of receptor-ligand docking and scoring, which can be used when a three-dimensional structure of the target is available. This method can be divided into two parts: first *docking *to position ligand structures into the target binding site, generating a set of poses for each ligand; and secondly *scoring *to evaluate and rank-order poses and ligands according to how well each pose for each ligand fits into the binding site and the quality of the interactions it forms with the target. Generally, in these methods the ligand has conformational flexibility while the receptor remains essentially rigid. The output from the docking program is thus a set of poses saved for each ligand, with a numerical score for each pose.

The main problem with virtual screening is that many, and in some cases the vast majority, of the compounds that are predicted to be active are in fact not active when screened experimentally. There are two theories found in the literature on the reasons for this phenomenon. Some researchers argue that docking can usually generate good poses of a ligand in an active site, however scoring functions are generally not successful at correctly ranking either ligands or poses [5]. Others have pointed out that docking programs do not always generate correct poses, and that the highest ranked pose for a given ligand is often incorrect. Scoring functions would therefore function much better at ranking if docking programs did not produce so many incorrect poses for each compound [6,7].

Regardless of whether it is the docking programs or the scoring functions that are at fault, the issue is that virtual screening can generate an enormous number of false positives – compounds that are scored highly *in silico *but do not actually bind to the target *in vivo *or *in vitro*. These false positives can also be blamed to some extent for false negatives, in the case where true positives are scored relatively poorly (and perhaps even eliminated) because of spuriously high-scoring false positives that are ranked ahead of them. A method of eliminating at least some of these false positives at some stage in the virtual screening procedure would therefore be very useful.

Here we present such a method, which we call pharmacophore filtering. It is a means of post-processing docking results to rapidly eliminate poses and molecules that are not fully chemically compatible with the binding site. This includes, for example, poses that do not completely fill the site, or that leave unpaired buried hydrogen bond donors or acceptors. This method could be viewed as an enforcement of the basic principle of structure-based drug design, namely that good-binding ligands must be chemically complementary to their receptors.

The advantages of including information about the target, such as specific, required hydrogen bonds into docking simulations is already well-appreciated [8]. One advantage of our method over others is that it allows multiple such target-ligand interactions to be quickly tested, compared and re-adjusted without re-running the entire docking calculation. Thus it is a useful addition to the virtual screening arsenal, and as we attempt to demonstrate here, it is broadly applicable to a variety of targets and ligand design goals.

## Results

### Pharmacophore Filtering Method Description

The pharmacophore filtering method begins by using a docking program in the ordinary way, for pose generation and alignment in the binding site. All the poses output by the docking program are saved to a file, while their scores and rankings are ignored. Next, the docked poses are searched and filtered using a series of pharmacophore query models. The pharmacophores are elucidated based on available crystal structures of bound ligands and/or simple examination of the binding site, and are used to filter the saved poses output from the docking program, and remove any that are incompatible with the model. This method thus adds an element of ligand-based drug design to what is essentially a structure-based drug design method of docking and scoring.

**Figure 1:**
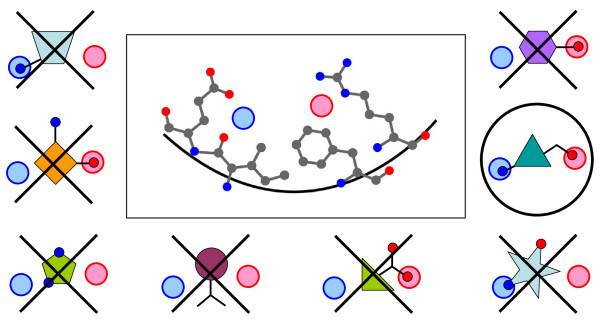
**A cartoon illustration of the pharmacophore filtering method**. A hypothetical binding site is shown in the central box, with interaction site points for a hydrogen bond donor (blue circle), and a hydrogen bond acceptor (red circle). A hypothetical set of docked poses is shown around the edges, superimposed on the interaction sites. The only pose which passes the filters and fits the binding site is circled while the others are crossed out.

Figure 1 gives a cartoon illustration of this methodology. In the center is shown a simple four-residue binding site. A ligand that binds well to this site must have a hydrogen bond donor group in the region marked with a blue circle in order to interact with the glutamate residue and the backbone carbonyl, and a hydrogen bond acceptor group in the region marked with a red circle to interact with the arginine residue. The set of saved poses from the docking program, shown around the edges of the figure, is then compared with this model for good binding. Unlike a traditional pharmacophore search, the ligands do not need to have low-energy conformers generated, or be translated or rotated to align to the pharmacophore hypothesis because they have *already *been aligned to one another in the coordinate space of the binding site by the docking program. Thus it is computationally inexpensive to step through the poses and check them against the pharmacophore model, and quickly eliminate those that do not fulfill the necessary interactions.

Clearly this method works best when at least one co-crystal structure with the natural ligand or an inhibitor is available, but it would also in theory be possible to use an apo structure and examine the interactions made with water. There are no special restrictions on what software to use, though it is probably better if the docking program has a stochastic component and can generate diverse poses rather than converging on a "best solution." In the test cases for this method, described below, we used the well-known docking programs GOLD [9] and Glide [10].

Rather than using a full-blown pharmacophore generation and search program, it is possible to simply filter the output file of docked poses according to whether or not they fulfill certain receptor-ligand contacts or interactions. For example, with Glide, Schrödinger provides a "Pose-Filter" Python script to perform this type of filtering, and for GOLD, such an analysis can be done with its companion programs Hermes and GoldMine. However, a dedicated pharmacophore program will provide more options and greater flexibility in defining the filters to be used.

The pharmacophore program must allow the import of a set of pre-generated conformers that are pre-aligned to the pharmacophore model. The site points in the pharmacophore model can be defined based on either the positions of ligand atoms (ligand-sided) or the positions of receptor atoms (protein-sided), or both, depending on the nature of the interaction to be captured with the filter. The radius of the site points can also be adjusted to alter the sensitivity of the model. A smaller radius gives a tighter, more selective filter, whereas a larger radius can capture some flexibility of ligands within the binding site or account for some variability in binding modes. For our test cases the pharmacophore models were generated in MOE [11] by simple visual inspection of crystal structure binding sites and co-crystallized ligands, along with information on the binding modes of other known ligands from the literature.

Alternately, there are several published methods and software programs available for automatically or semi-automatically generating structure-based pharmacophore models. For example, the program LUDI [12] (currently commercially available as part of Discovery Studio from Accelrys) calculates an interaction map of locations in the receptor binding site where an atom from a bound ligand would be in position to form a favorable hydrogen-bonding or hydrophobic interaction. This map can then be converted into a pharmacophore model. Another such program is LigandScout [13], which is a fully automated method of generating a pharmacophore model from a set of protein-ligand complexes.

### Test Cases

We evaluated our methodology with three test cases: protein targets of pharmaceutical relevance with a variety of binding site characteristics.

#### Neuraminidase A

Influenza neuraminidase A is a surface glycoprotein of the influenza virus whose function is to cleave the linkage between sialic acid and an adjacent sugar in glycoconjugates on the surface of cells targeted for infection [14]. The sialic acid binding site is small, deep, and highly polar. For this target, there existed a literature reference examining which of the many available crystal structures of neuraminidase is best suited for docking a variety of ligands [15]. Based on this study's conclusions, we chose the 1MWE crystal structure [16], which has good (1.7 Å) resolution and could accommodate all members of a set of co-crystallized ligands [15]. To prepare the structure for docking, we deleted all bound ligands and crystallographic waters and flipped the orientation of residue Asn 294 in the binding site (the sidechain amide nitrogen and oxygen had been assigned incorrectly).

The screening database for this test case was constructed by first downloading a set of 245 neuraminidase ligands from the BindingDB [17,18]. Ligands with K_i _or IC_50 _values of 10 μM or lower (195 compounds) were considered actives. The inactive ligands were added to a set of 4775 decoys obtained from both the MDL Drug Data Report database (MDDR) [19] and the ChemNavigator iResearch Library (iRL) [2] of commercially available compounds. The decoys were filtered using Pipeline Pilot [20] to choose compounds whose physicochemical properties fit into the ranges seen with the known neuraminidase ligands. This was to ensure that the docking and scoring protocols were not biased in distinguishing hits from decoys due to differences in their property distributions. In this case, decoys were required to have a molecular weight between 190 and 500, a logP between -7.5 and 4.0, a polar surface area between 95 and 250 Å^2^, fewer than 10 rotatable bonds, at least 3 hydrogen bond donors and at least 2 hydrogen bond acceptors. Thus the final set of 4775 decoys were all as small and highly polar as the set of known neuraminidase binders.

The screening database was docked into the binding site using two docking programs, GOLD 3.0.1 [9] and Glide 4.0 [10]. In GOLD, the binding site was defined as a sphere with a radius of 10 Å centered at the position of atom C6 in the bound sialic acid ligand. We used the 7–8x speedup rather than the library screening settings for the genetic algorithm, for some sacrifice in speed but an improvement in the quality of the generated poses, and the Goldscore scoring function. In Glide, the protein structure was prepared using the Protein Preparation and Grid Generation modules, with all default settings. For docking we used the HTVS precision mode, with all other parameters left in their default settings. With both programs, ten poses were saved for each compound, then all the saved poses from both programs were submitted to pharmacophore filtering (see below). For comparison purposes, all the saved poses from both programs were also re-scored with a total of four different scoring functions: GScore in Glide, Goldscore and Chemscore as implemented in GOLD, and an "Affinity" score. This latter score is a reformulation of two of the terms in the Goldscore scoring function, defined as *hbond.external *+ 1.375*vdw.external, and has been shown to correlate better with experimental binding affinities than Goldscore itself, which was optimized to give good poses [21].

**Figure 2:**
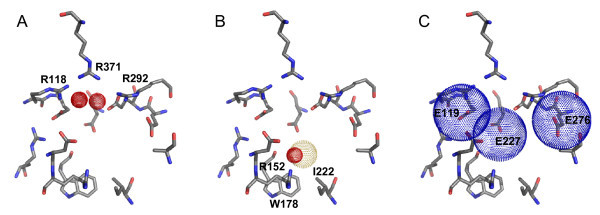
**The sialic acid binding site and the three pharmacophore filters defined for neuraminidase A**. A) Site points, shown as red-dotted spheres, indicate the required positions of hydrogen bond-accepting or negatively charged atoms to interact with arginine residues in the acid binding sub-pocket. B) The required position of an atom forming a hydrogen bond to Arg 152 (red-dotted sphere) along with a hydrophobic interaction (yellow-dotted sphere). C) Blue-dotted spheres indicate the space available for atoms forming hydrogen bonds or salt bridges to three glutamate residues. This figure was generated using PyMOL [22].

The pharmacophore filters used for neuraminidase are shown relative to the binding site in Figure 2[22]. To develop these filters we first reproduced the docking of a set of 21 co-crystallized ligands back into the 1MWE crystal structure, as was done by Birch et al. in their analysis [15]. This set of superimposed docked ligands along with the binding site residues were then imported into the pharmacophore query editor in MOE [11]. All of the ligands contain a carboxylic acid or phosphate group that interacts with three strictly conserved arginine residues (Arg 118, 292, and 371) [14] in the so-called acid binding sub-pocket of the binding site. Since the charged oxygens in these acidic groups were tightly superimposed, we built two ligand-sided site points (meaning that they were centered over the positions of the superimposed ligand atoms) on these oxygens, as shown in Figure 2A. This created the first filter, to select for docked poses in the screening database with hydrogen bond acceptor atoms or negatively charged atoms in position to interact with the arginine residues.

Another sub-pocket of the binding site holds an acetyl group via a hydrogen bond from the carbonyl oxygen to Arg 152, and a hydrophobic interaction of the methyl group with residues Ile 222 and Trp 178 [14]. We constructed a second filter for this interaction with a ligand-sided hydrogen bond acceptor or anionic site point and a ligand-sided hydrophobic site point (Figure 2B).

Finally, the third filter took into consideration the three highly conserved glutamate residues with unpaired acceptor atoms across the center of the binding site (Glu 119, 227, and 276) [14]. The superimposed co-crystal ligands showed a variety of methods of interacting with these glutamates, so we built receptor-sided site points (meaning that they were centered on oxygen atoms in the glutamate residues of the receptor) for hydrogen bond donor or positively charged atoms, with a radius of about 3 Å to allow for the hydrogen bonding distance to ligand atoms (Figure 2C). The filter required an interaction with at least one of the glutamates.

**Figure 3:**
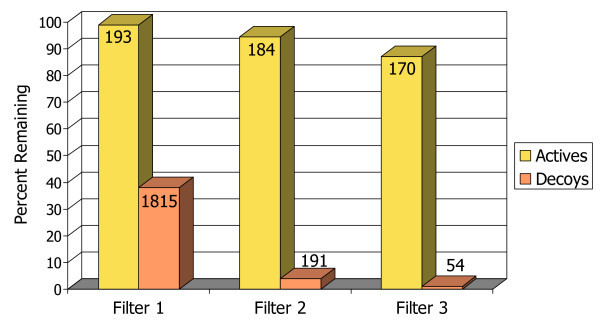
**Bar chart illustrating filtering rates for actives vs. decoys with neuraminidase A**. The percentages of actives (true positives) and decoys (false positives) that remain in the docked database after each of the pharmacophore filters. The absolute number in each group of compounds is marked at the top of the bars.

The bar chart in Figure 3 illustrates schematically the ability of each of the three pharmacophore filters, applied sequentially, to selectively remove false positives (decoys) out of the set of docked poses while retaining true positives (known actives). The three filters dramatically reduce the number of decoys down to approximately 1% of their original number, while keeping nearly 90% of the true positive compounds. By the last filtering step, the starting set of 4970 compounds has been reduced to only 224, which might be a reasonable number for a small-scale experimental screen. This final hit set has about three times as many true actives as decoys.

**Figure 4:**
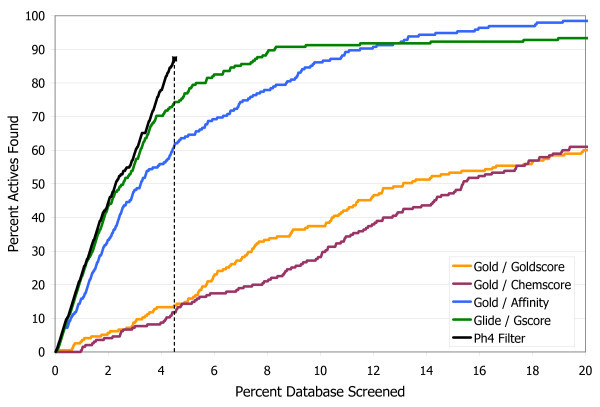
**Enrichment plot for neuraminidase A**. Comparison of the percentage of known actives retrieved vs. the percentage of the test database screened for the pharmacophore filtering method (black line) and traditional docking + scoring methods.

To compare these results to the traditional scoring function approach, in Figure 4 the final hit set after filtering has been ranked numerically by docking score, and is plotted on a standard enrichment plot (percentage of actives found vs. percentage of database screened), along with the original set of all the docked compounds ranked by each of the four scoring functions. Neuraminidase is not a target that is particularly challenging for most docking programs, as illustrated by the fact that both Glide/Gscore and Gold/Affinity are able to rank over 90% of the actives in approximately the first 10% of the database. Nevertheless there is still a clear improvement in enrichment with the pharmacophore filtering. Another point that can be highlighted here is that while it is often difficult to know with scoring functions what cutoff to use for delineating good scores from bad scores, or good compounds from bad compounds, with the pharmacophore filtering method an automatic cutoff is built in, as shown by the dashed line in Figure 4 indicating the point beyond which no structures had any poses that passed all three filters in the pharmacophore model. This eliminates over 95% of the starting database from further consideration. Thus instead of requiring the somewhat arbitrary selection of the top N molecules from a list ranked by docking score, the pharmacophore filtering method produces the clear result that 224 compounds are worthy of further consideration, either with experimental screening or with more detailed modeling work.

#### Cyclin-dependent kinase 2 (CDK2)

The family of cyclin-dependent kinases are regulators of the cell cycle. In particular CDK2, in complex with cyclins E and A, is involved in the G1-S transition and the progression through S phase, although it has recently been shown not to be essential for mitotic cell division in mice [23]. Like all other kinases, CDK2 has an ATP binding site located in a cleft between the N-terminal and C-terminal lobes of the protein. Here again, as with neuraminidase, we consulted the literature to determine which of the available crystal structures would be best suited for virtual screening. There is a published study examining a diverse subset of 20 of the available CDK2 crystal structures, and we chose the one that performed best at docking the most structurally diverse group of CDK2 inhibitors [24]. This crystal structure, 1OIT, is in an active, open conformation and has a high crystallographic resolution of 1.6 Å [25]. Several loop regions in the structure were disordered and coordinates for these were not present. We capped all such protein chain ends, including the N- and C-termini, with hydrogens to form neutral NH_2 _and COOH groups. We also built coordinates for the missing sidechain of residue Lys 9, which is near the binding site, in a conformation pointing out into solvent with a χ^1 ^angle of -60°.

Along the same lines as with neuraminidase, the screening database was constructed by first downloading a set of 1278 CDK2 ligands from the BindingDB [17,18]. Ligands with K_i _or IC_50 _values of 10 μM or lower (1063 compounds) were considered active, and the inactive ligands were added to a set of 26792 decoys combined from the MDDR [19] and ChemNavigator iRL [2] databases. These decoys were filtered using Pipeline Pilot [20] to choose compounds physicochemically similar to known kinase inhibitors, with a molecular weight between 200 and 550, at least two aromatic rings, a logP between 0.5 and 6.0, a polar surface area of less than 150 Å^2^, fewer than 10 rotatable bonds, less than 4 hydrogen bond donors and between 2 and 8 hydrogen bond acceptors.

The screening database was docked into the binding site using both GOLD [9] and Glide [10], with the same settings used as before with neuraminidase. In GOLD, the binding site was defined as a sphere with a radius of 10 Å centered at the position of atom C14 in the bound ligand. Ten docked poses were saved for each compound from each program, and all the poses were again submitted to pharmacophore filtering. All the saved poses were also re-scored as before with the four scoring functions: GScore, Goldscore, Chemscore, and GOLD Affinity.

**Figure 5:**
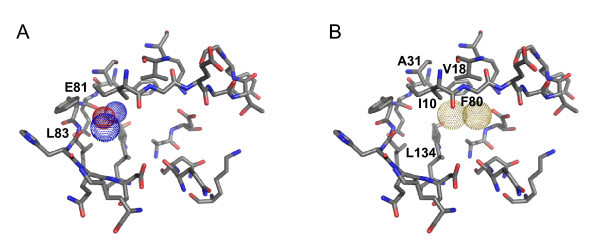
**The ATP binding site and the two pharmacophore filters defined for CDK2**. A) The required locations for hydrogen bond-donating atoms (blue-dotted spheres) and a hydrogen bond-accepting atom (red-dotted sphere) in the hinge region. B) Overlapping yellow-dotted spheres delineating the space to be filled by hydrophobic packing in the main pocket of the binding site. This figure was generated using PyMOL [22].

The pharmacophore filters used for CDK2 are shown in Figure 5[22]. To develop these filters, we proceeded in a similar way as for neuraminidase and docked a small set of 12 co-crystallized CDK2 ligands back into the 1OIT binding site [24]. The set of superimposed docked ligands along with the binding site residues were then imported into the pharmacophore query editor in MOE [11]. Both pharmacophore filters for this target used ligand-sided site points. The first filter (Figure 5A) was constructed to select for poses with hydrogen bonds to the hinge region of the binding site, a standard kinase inhibitor feature. There are three possible hydrogen bonds that can be formed, to Leu 83 NH, Leu 83 O, or Glu 81 O. The filter required at least two out of three of these to be present. The second filter (Figure 5B) selected for ligands of the right size and shape to fill the hydrophobic adenine region of the binding site, interacting with residues Phe 80, Val 18, Ile 10, Leu 134, and/or Ala 31.

**Figure 6:**
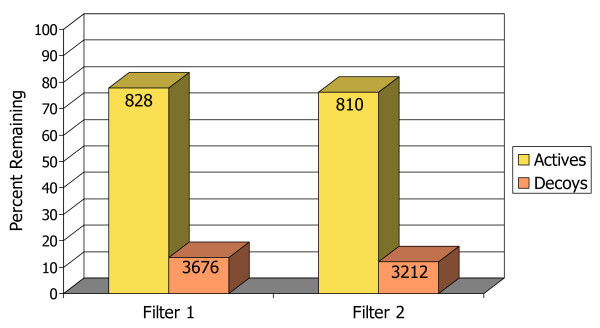
**Bar chart illustrating filtering rates for actives vs. decoys with CDK2**. The percentages of actives (true positives) and decoys (false positives) that remain in the docked database after each of the pharmacophore filters. The absolute number in each group of compounds is marked at the top of the bars.

The bar chart in Figure 6 illustrates how well these two pharmacophore filters selected for true positives (known actives) over false positives (decoys) in the set of docked poses. Here the filtering is not as successful at separating actives from decoys as with neuraminidase, but the number of decoys is reduced down to about 10% of the starting number, while over 75% of the true active compounds are retained.

**Figure 7:**
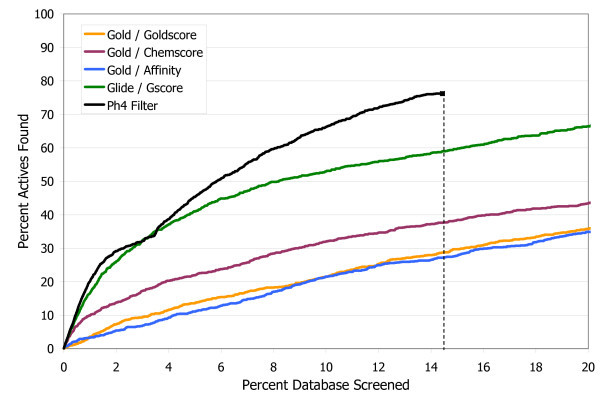
**Enrichment plot for CDK2**. Comparison of the percentage of known actives retrieved vs. the percentage of the test database screened for the pharmacophore filtering method (black line) and traditional docking + scoring methods.

The enrichment in the pharmacophore-filtered poses (Figure 7) is again improved over the best-performing scoring function, which is Glide/GScore. As before, the pharmacophore filtering creates a natural cut-off point (shown by the dashed line in Figure 7) for separating good compounds from bad compounds. Less than 15% of the full database passes both filters, and all other compounds can be concluded to be either incompatible with the binding site, or to have been misdocked, and therefore can be safely ignored.

**Figure 8:**
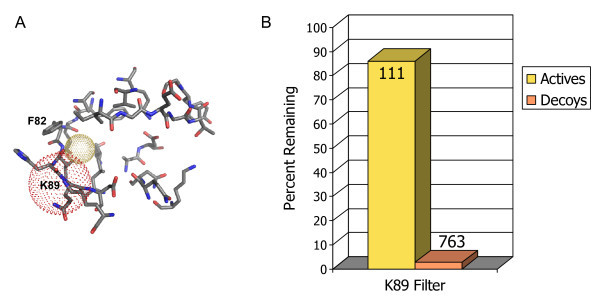
**Secondary pharmacophore filter for CDK2**. A) The ATP binding site with specificity-conferring interactions: the position of an atom accepting a hydrogen bond from Lys 89 (red-dotted sphere) and a hydrophobic interaction (yellow sphere). This figure was generated using PyMOL [22]. B) Bar chart showing the percentage of hits (known actives) compared to decoys that are left in the test database after this pharmacophore filter is applied. The absolute number in each group of compounds is marked at the top of the bars.

To further refine these results, we looked for subsets of compounds in the set of CDK2 ligands from the BindingDB that exploit a particular non-conserved region in the active site to give selectivity for CDK2 over other kinases and other members of the CDK family. Some CDK2 ligands that have a sulfonamide group that is positioned to interact with a lysine residue (Lys 89) on the top edge of the binding site, adjacent to a phenyl ring (Phe 82) that packs into a small secondary hydrophobic pocket or slot above the hinge region (Figure 8A). These ligands include oxindole-based compounds [26], imidazo [1,2-*a*]pyridines [25], and imidazo [1,2-*b*]pyridazines [27]. There were 129 molecules in the set of CDK2 ligands that fit this profile. A final pharmacophore filter was set up in MOE [11] to look specifically for compounds that can make these interactions that confer CDK2 specificity (Figure 8A). This filter was built with a protein-sided acceptor or anionic site point centered on the Nζ atom of Lys 89, and a hydrophobic or aromatic site point positioned to interact with residues Ile 10 and Phe 82. With this filter, we were able to extract nearly 90% of the 129 active compounds in the subset, and the number of decoys was reduced to less than 3% of their starting number (Figure 8B).

#### Protein kinase C (PKC) C1 domain

Protein kinase C isozymes are a family of serine/threonine kinases which are centrally involved in cell signaling. The regulatory C1 domain is a 50-residue zinc-finger-like structure that responds to the second messenger diacylglycerol (DAG) by translocating PKC to the cell membrane, where the whole protein undergoes a conformational change and the kinase domain becomes activated [28]. This test case was a more difficult target. The natural activator is a lipid, and other known ligands are all also lipophilic natural products [29] and derivatives thereof [30]. Furthermore, the binding site in the C1 domain is a half-site in that ligands make interactions with the C1 domain itself, but interactions with lipids in the cell membrane are also important for binding [31]. There is only one holo C1 domain crystal structure (1PTR) available in the PDB, with phorbol ester (a natural tumor promoter and DAG mimetic) in the binding site [32].

The screening database consisted of a small collection of 27 known C1 domain ligands [29,30,33], along with a set of 1096 natural product decoys from several databases: the Natural Products subset of the Open NCI Database [34], a database of compounds used in traditional Chinese medicine [35], and a database of natural products isolated from marine species [36]. As with the other two test cases, we selected decoys using Pipeline Pilot [20] that were physicochemically similar to the known ligands. Decoys were required to have a molecular weight greater than 250, a logP between 0.5 and 6, at least three hydrogen bond acceptors and at least one hydrogen bond donor, less than 250 Å^2 ^of polar surface area, and more than 220 Å^2 ^of non-polar surface area. High-quality three-dimensional structures for these often-complex molecules were generated using CORINA [37].

The screening database was docked, as before, with GOLD [9] and Glide [10]. Due to the fact that the natural products tended to be large compounds with many rotatable bonds, we used standard precision docking in Glide and the default genetic algorithm settings in GOLD rather than high-throughput or speeded-up screening settings, and we increased the sampling by saving 20 poses for each compound. In GOLD, the binding site was defined as a sphere with a radius of 10 Å centered at the position of the Nε atom in residue Gln 257. All saved poses were submitted to pharmacophore filtering, and also re-scored as before with the four scoring functions: GScore, Goldscore, Chemscore, and GOLD Affinity.

**Figure 9:**
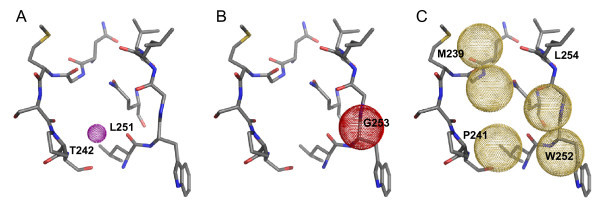
**The C1 domain diacylglycerol binding site and the three pharmacophore filters defined for PKC**. A) The required position of a hybrid donor/acceptor group (purple-dotted sphere). B) The space available for a hydrogen bond-accepting atom forming a key interaction with Gly 253 (red-dotted sphere). C) Hydrophobic groups (yellow-dotted spheres) in position to interact with the lipid bilayer. This figure was generated using PyMOL [22].

The pharmacophore filters used are shown in Figure 9[22] and were based on the hydrogen bonding interactions seen between the co-crystallized phorbol ligand and the C1 domain binding site [32]. First (Figure 9A), we used a ligand-sided site point to select for poses with a functional group at the bottom of the binding site acting as both a donor and an acceptor, and forming hydrogen bonds to a backbone carbonyl oxygen on one side of the binding site (Leu 251) and to a backbone nitrogen on the other side of the binding site (Thr 242). Secondly (Figure 9B), we looked for poses that could form a hydrogen bond to a glycine residue (Gly 253) on the outer edge of the binding site, via a protein-sided acceptor site point centered on its backbone N atom. These two interactions are believed to be conserved across all known C1 domain ligands [29,38]. The final filter simply selected for poses with a certain amount of hydrophobic bulk located in the region above the binding site, that would be in position to interact with the lipid membrane and with hydrophobic residues around the edge of the site. At least two of the five site points shown in Figure 9C were required by the filter.

**Figure 10:**
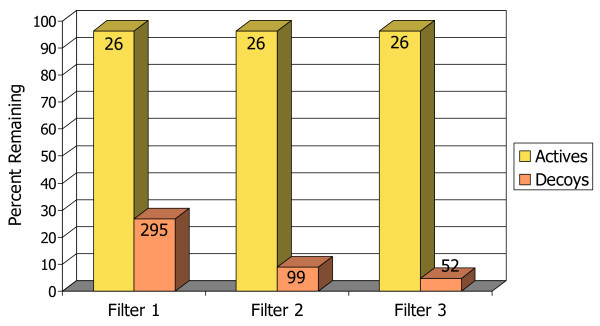
**Bar chart illustrating filtering rates for actives vs. decoys with PKC**. The percentages of actives (true positives) and decoys (false positives) that remain in the docked database after each of the pharmacophore filters. The absolute number in each group of compounds is marked at the top of the bars.

**Figure 11:**
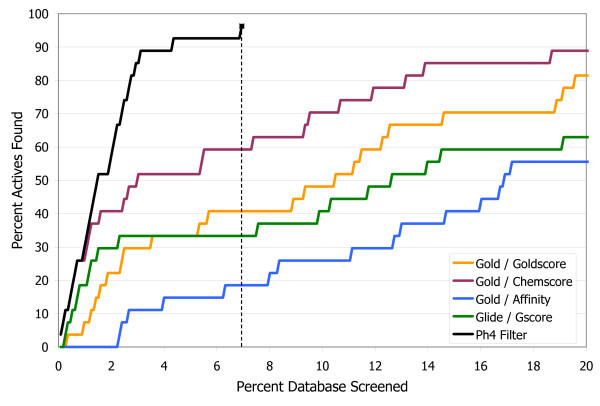
**Enrichment plot for PKC**. Comparison of the percentage of known actives retrieved vs. the percentage of the test database screened for the pharmacophore filtering method and traditional docking + scoring methods.

The performance of the pharmacophore filters is shown in the bar chart in Figure 10. Of the original set of 27 known ligands all but one passed through all three filters, and that compound had 22 rotatable bonds and so it was misdocked as both docking programs had difficulty with it. The number of decoys was reduced to about 5% of the starting number. The enrichment plot, Figure 11, shows that the pharmacophore filtering method performed substantially better with this target than any of the traditional scoring functions. This is probably due to the hydrophobic nature of the binding site and the relatively small number of polar interactions. The cut-off point generated by the pharmacophore filtering (the dashed line in Figure 11) gives a final hit set of only 78 compounds.

## Discussion

In all three of the test cases at least one crystal structure of the target and a collection of known inhibitors were available, allowing us to use properties calculated for known inhibitors to make rational decisions on the ranges of values for physicochemical properties that are reasonable for new potential inhibitors of the target. We used the structural characteristics of the binding site and the observed interactions between target and ligands to choose the parameters used in docking and scoring in order to reproduce known inhibitor binding modes, and for analysis of the docked poses with the pharmacophore filtering method. We have shown here that, compared to simply ranking the poses output by the docking program according to a single docking score, an initial filtering of the docked poses with a pharmacophore query to remove poses that do not form certain essential interactions with the target binding site greatly improves the quality of the results. Typically we have seen the ability to eliminate well over 90% of the decoy molecules in the starting database while retaining about 80% of the true positives.

This pharmacophore filtering method is in many ways similar to other approaches that attempt to add additional information to improve the results from docking and scoring. We will briefly delineate them, and then discuss what we believe are the advantages of the method presented here. One alternative way to incorporate the use of pharmacophores into a virtual screening protocol is to use them for pre-screening, rather than post-screening, the docking database. Such a strategy has been used successfully with several targets [39,40], and has the advantage of reducing the size of the database to be docked. A variation on this method is called pharmacophore-based molecular docking, or PhDOCK [41], as it is a variation of the DOCK program. A database of conformers is overlaid according to 3D pharmacophores and the pharmacophore site points are then matched with docking site points in the binding site. This allows the simultaneous docking of many molecules at once. The docking site points can either be generated from standard DOCK "spheres" or can be based on the positions of atoms in crystallographic ligands. The advantage of these pre-screening methods is that they can significantly shorten the amount of computer time required for docking, though this becomes less and less relevant as computer speed increases and computer clusters become more common.

A second method of incorporating pharmacophores into virtual screening is to post-process docking results with various kinds of ligand interaction-based filters. One such method is the structural interaction fingerprint [42], or molecular interaction fingerprint [43], which encodes into a binary bit string the set of binding interactions made by a docked ligand pose. These strings can be clustered and compared using Tanimoto similarities, and used to rapidly filter poses for the presence or absence of specific interactions, providing an improvement in virtual screening hit list enrichment over conventional scoring functions. Similarly, an interactions-based accuracy classification method [44] has been shown to provide a superior assessment of docking pose quality compared to simple RMS deviations from crystal structure poses. Rather than applying pharmacophores to filter docked poses directly, the AutoShim method [45] uses point pharmacophore interaction features in the binding site to weight or "shim" the docking scoring function according to experimental IC_50 _data for the target receptor, and can produce large improvements in the ability of the scoring function to predict binding affinities. In contrast, the pharmacophore filtering method presented here requires no additional new or proprietary software unlike these other methods for post-processing docking results – only a docking program and a pharmacophore generation program, both of which are highly likely to be in use by any modeling group.

Finally, a third method of incorporating pharmacophores into virtual screening is to add constraints to the docking run to ensure that certain interactions between the ligands and the target binding site are formed. Such pharmacophoric constraints can be implemented in GOLD [46] as well as in many other modern docking programs, and are the basis for the screening program FlexX-Pharm [47], where ligands are incrementally constructed into the active site in a manner incorporating "look-ahead checks" to ensure that desired interactions are formed. These types of constraints are particularly useful with kinases, in which hydrogen bonds between ligands and the hinge region of the binding site are almost universally present [7]. Adding this kind of additional information to the docking has been shown to improve enrichment, though this may come at the expense of a reduction in ability to identify novel chemotypes [5].

Although we have not compared our method directly with these constrained docking methods, we would anticipate that the enrichments seen would be quite similar (if not better with constrained docking because the conformational search done by the docking program is biased in favor of solutions that fit the constraints). However, the great advantage of the pharmacophore filtering method is that it allows the modeler to change his or her mind about which features are most important for a productive binding interaction without having to go back and redo the docking runs. A typical pharmacophore filtering run on a file of 100,000 docked poses takes only a few minutes, whereas the docking runs themselves can take days or weeks. It is therefore possible to quickly test multiple filtering combinations and variations, and to develop refined hit sets focusing on different regions of the binding site.

It is also possible to adjust the stringency of the filters to tune the number of compounds that are output from the pharmacophore filtering, to be sent on to the next step in the screening. If the hit set is to be assayed experimentally, the number of compounds can be scaled according to the resources available and the throughput level of the assay. If more detailed calculations are to be done, such as MM/PBSA scoring or other free energy estimations of binding affinity, the size of the hit set can be adjusted according to the available computational resources and time.

A final advantage of our pharmacophore filtering method is the ability to easily go back and look for compounds occupying specific sub-pockets in the binding site, as for example with CDK2 and residue Lys 89 on the top edge of the binding site. It is also possible to explore novel binding modes, to look for compounds that interact with the target receptor in ways that are not seen with existing known ligands.

The three test cases presented here are of course not sufficient to provide an estimate of the statistical significance of the improvement in performance of this method over traditional docking + scoring methods, and in fact for two of the targets (neuraminidase and CDK2) the improvement seen with the pharmacophore filtering method versus Glide docking with compounds ranked by Gscore is very slight. However, there is no way to know *a priori *whether or not a given docking + scoring method will perform well for a given target. Many published studies comparing docking programs to one another have shown widely varying results for different targets [5,48,49]. In this study, although Glide performs very well with neuraminidase and CDK-2, GOLD is more successful with the third target, the PKC C1 domain.

We also believe that human oversight and intervention in any computational modeling work is essential. In virtual screening, time spent thinking carefully about the receptor binding site and interactions made by ligands binding in it would pay off in a greater understanding of the structural characteristics of the system – knowledge that could be useful for subsequent refinement into leads of the hits out of the screening database. Our rapid and resource-wise undemanding method facilitates this thoughtful approach to one of the challenges in drug design.

## Conclusion

In summary, combining docking with pharmacophore searches as a post-processing filter allows the advantages of both methods to be fully exploited. The docking program is used to fit the ligands into the binding site in as wide a variety as possible of reasonable ways. Put another way, we are using it as a conformation generator for conformations that fit into the active site. Compared to a traditional pharmacophore search, where conformations for each molecule in the database are pre-generated to be dispersed over all low-energy conformational space, the docking program allows us to focus on a set of conformations for each molecule that all fit into the binding site. Additionally, the docking program performs the alignment of all the conformations to one another, which is much simpler than dealing with the combinatorial explosion of ways in which the active compounds can be aligned and the number of features to include. Post-processing the docking results with pharmacophore filtering allows us to bypass to a large extent the difficulty with scoring functions, which is that while they are generally good at producing reasonable docked poses of a molecule in a binding site, they are not necessarily good at discriminating between good binders and poor binders, probably due to the non-linear nature of ligand-receptor recognition. The pharmacophore filtering method greatly increases the likelihood that the best and/or most correct pose is selected from the set of docked poses, regardless of the numerical value of its docking score. This method has been used successfully in several virtual screening projects in our laboratory, one for inhibitors of Met tyrosine kinase [50], and others which will be reported separately.

## Competing interests

The authors declare that they have no competing interests.

## Authors' contributions

MLP conceived of the method, carried out the computational work and data analysis, and drafted the manuscript. MCN implemented the resources necessary for the computational screening, supervised the work, and participated in the manuscript revisions. All authors read and approved the final manuscript.
